# Association Between DASH Diet Quality and 24 h Ambulatory Blood Pressure in Treatment-Naive Adults Referred for Diagnostic Monitoring: A Cross-Sectional Study

**DOI:** 10.3390/medicina62050974

**Published:** 2026-05-17

**Authors:** Nezihe Otay Lule, Mert Deniz Savcilioglu, Kemal Ozan Lule, Mehmet Murat Sucu

**Affiliations:** 1 Nutrition and Dietetics Department, Faculty of Health Sciences, Gaziantep University, 27310 Gaziantep, Türkiye; 2 Department of Cardiology, Faculty of Medicine, Gaziantep University, 27310 Gaziantep, Türkiye; mdsavcilioglu@gmail.com (M.D.S.); mmuratsucu@gmail.com (M.M.S.); 3 Internal Medicine Department, Faculty of Medicine, Gaziantep University, 27310 Gaziantep, Türkiye; drkemalozanlule@gmail.com

**Keywords:** DASH diet, ambulatory blood pressure monitoring, hypertension, diet quality, DASH-Q questionnaire, blood pressure

## Abstract

*Background/Objectives*: Dietary adherence to the Dietary Approaches to Stop Hypertension (DASH) pattern is associated with lower blood pressure; however, most prior studies have relied on office-based measurements and non-specific dietary assessment tools. This study examined the association between DASH diet quality, assessed by the validated DASH-Q questionnaire, and 24-h ambulatory blood pressure in treatment-naive adults referred for diagnostic ambulatory blood pressure monitoring (ABPM). *Materials and Methods*: This cross-sectional study enrolled 227 consecutive treatment-naive adults referred for diagnostic 24-h ABPM at a cardiology outpatient clinic. DASH diet quality was assessed using the validated Turkish version of the DASH-Q questionnaire and categorized as low (<36), moderate (36–49), or high (≥50). Hypertension was defined by ABPM-based thresholds. Multivariable linear regression was performed to identify independent predictors of 24-h mean systolic and diastolic blood pressure, and binary logistic regression was used to evaluate independent predictors of ABPM-defined hypertension, with both models adjusted for age, sex, BMI, smoking, physical activity, and self-reported discretionary salt-adding behavior. *Results*: DASH-Q total score was the sole statistically significant independent predictor of both 24-h mean systolic blood pressure (B = −1.068, 95% CI: −1.270 to −0.866; β = −0.589; *p* < 0.001) and diastolic blood pressure (B = −0.560, 95% CI: −0.706 to −0.414; β = −0.470; *p* < 0.001) in the adjusted models. Each one-unit higher DASH-Q score was also associated with 14.6% lower odds of ABPM-defined hypertension (OR = 0.854, 95% CI: 0.820–0.890; *p* < 0.001). Higher DASH-Q scores were further associated with a more favorable metabolic profile, including lower LDL cholesterol, triglycerides, glucose, and C-reactive protein levels. *Conclusions*: DASH diet quality was independently and inversely associated with 24-h ambulatory blood pressure and the odds of ABPM-defined hypertension in this treatment-naive population. Given the cross-sectional design and the possibility of reverse causality, these results should be interpreted as hypothesis-generating and require confirmation in prospective studies.

## 1. Introduction

Hypertension remains one of the leading modifiable risk factors for cardiovascular morbidity and mortality worldwide, affecting an estimated 1.28 billion adults globally [1,2]. Despite substantial advances in pharmacological treatment, blood pressure control rates remain unsatisfactory in many populations [3]. Accordingly, contemporary guidelines continue to emphasize lifestyle modification, including dietary intervention, as a fundamental component of blood pressure prevention and management [4,5,6].

The Dietary Approaches to Stop Hypertension (DASH) diet is among the most extensively studied dietary strategies for blood pressure reduction. In the landmark DASH trial, a dietary pattern rich in fruits, vegetables, whole grains, and low-fat dairy products, with reduced saturated fat intake, significantly lowered blood pressure in both hypertensive and normotensive adults [7]. The DASH-Sodium trial subsequently demonstrated additional blood pressure reduction when the DASH dietary pattern was combined with sodium restriction [8]. Since then, systematic reviews and meta-analyses have consistently confirmed the blood pressure-lowering effects of the DASH diet across different populations and clinical settings [9,10,11,12].

However, most studies examining DASH adherence and blood pressure have relied on office-based measurements, which are subject to white-coat effects, masked hypertension, and substantial short-term variability [13,14]. By contrast, 24-h ambulatory blood pressure monitoring (ABPM) provides a more comprehensive assessment of blood pressure burden over the full circadian cycle, including daytime and nighttime values, and has demonstrated superior prognostic value compared with office blood pressure measurement [13,15,16]. For this reason, current guidelines recommend out-of-office blood pressure assessment, particularly ABPM, for the diagnosis and risk stratification of hypertension [4,5,17].

Assessing adherence to the DASH diet in observational studies also requires practical and validated instruments. The DASH Quality (DASH-Q) questionnaire is a brief, 11-item self-report measure developed and psychometrically validated to assess DASH diet adherence in adults [18]. A validated Turkish version is available, making it suitable for use in Turkish populations [19]. Compared with broader dietary assessment tools, the DASH-Q offers a focused and feasible approach to capturing overall DASH dietary adherence in routine clinical and research settings.

Despite the strong evidence supporting the DASH diet, studies specifically evaluating DASH diet quality with a validated DASH-oriented instrument in conjunction with 24-h ABPM remain scarce. Most available studies have assessed dietary patterns using food frequency questionnaires and blood pressure using office measurements rather than ambulatory monitoring [20,21]. Furthermore, whether DASH diet quality is independently associated with the likelihood of ABPM-defined hypertension as a categorical clinical outcome has not been adequately examined in treatment-naive populations referred for diagnostic evaluation.

Therefore, the present study had three primary aims: (1) to examine the associations between DASH diet quality, assessed by the DASH-Q questionnaire, and 24-h ambulatory systolic and diastolic blood pressure as continuous outcomes; (2) to determine whether these associations remained statistically significant after adjustment for major demographic, anthropometric, and lifestyle-related covariates using multivariable linear regression; and (3) to evaluate whether DASH diet quality was independently associated with the odds of ABPM-defined hypertension using binary logistic regression. We hypothesized that higher DASH-Q scores would be associated with lower 24-h ambulatory blood pressure and with reduced odds of ABPM-defined hypertension, independently of major confounders.

## 2. Materials and Methods

### 2.1. Study Design and Population

This was a cross-sectional study conducted at the Cardiology Outpatient Clinic of Gaziantep University Şahinbey Research and Practice Hospital, Gaziantep, Turkey. A total of 227 treatment-naive adults referred for diagnostic 24-h ambulatory blood pressure monitoring (ABPM) were consecutively enrolled. All participants were being evaluated for a suspected new diagnosis of hypertension and were not receiving antihypertensive medication at the time of enrollment. Referral for ABPM was based on clinical suspicion of hypertension rather than dietary status. In routine cardiology practice, patients were referred for diagnostic ABPM when they had borderline or repeatedly elevated office and/or home blood pressure readings, cardiovascular or metabolic risk factors requiring more accurate blood pressure characterization, or suspected masked hypertension despite non-diagnostic office measurements. DASH-Q scores were obtained after referral and were not used in the decision to perform ABPM.

Participants were eligible if they were aged 18 years or older, were referred for diagnostic 24-h ABPM, and provided written informed consent. Exclusion criteria included secondary causes of hypertension, chronic kidney disease (estimated glomerular filtration rate < 30 mL/min/1.73 m^2^), known malignancy, pregnancy, current use of antihypertensive medications, and use of medications known to substantially affect blood pressure (e.g., systemic corticosteroids or oral contraceptives). Participants with technically inadequate ABPM recordings (<80% valid readings) were also excluded.

This study was conducted in accordance with the Declaration of Helsinki and approved by the Gaziantep University Non-Interventional Clinical Research Ethics Committee (Number:2026/142, Date: 18 February 2026). Written informed consent was obtained from all participants prior to their inclusion in the study. The study followed the STROBE reporting guidelines.

### 2.2. Sample Size and Power Analysis

The sample size was determined on the basis of the study’s three primary hypotheses, using effect sizes derived from the cross-sectional study by Harrington et al. [22]. Power analyses were performed for each hypothesis, and the largest required sample size was 114 participants for the adjusted association analysis. To account for potential data loss related to ABPM failure, incomplete questionnaire data, and other exclusions, a larger sample was targeted. With the final sample of 227 participants, the study achieved >80% statistical power for all three primary hypotheses.

### 2.3. Assessment of DASH Diet Quality

Diet quality was assessed using the validated Turkish version of the DASH Quality (DASH-Q) questionnaire [18,19]. The DASH-Q is a self-administered, 11-item instrument that evaluates the frequency of consumption of key DASH-related food groups during the preceding 7 days. Total scores range from 0 to 70, with higher scores indicating better adherence to the DASH dietary pattern. Based on the original validation study, participants were categorized as having low diet quality (<36), moderate diet quality (36–49), or high diet quality (≥50).

### 2.4. Ambulatory Blood Pressure Monitoring

Twenty-four-hour ambulatory blood pressure monitoring (ABPM) was conducted using a validated oscillometric device (Borsam Echo BP Holter^®^, Borsam Biomedical Instrument Co., Shenzhen, China). Blood pressure was recorded automatically at 15-min intervals during the daytime and 30-min intervals during the nighttime. Participants were instructed to continue their usual daily activities and to keep the monitored arm still during each measurement. Daytime and nighttime periods were determined based on participants’ self-reported wake and sleep times. Mean daytime and nighttime systolic and diastolic blood pressure values were calculated automatically using the device software (eCardio Holter Analysis Software, version 7.0; Borsam Biomedical Instruments Co., Ltd., Shenzhen, China). Recordings with fewer than 80% valid measurements were excluded from the analysis.

Hypertension was defined according to 24-h ABPM thresholds recommended in contemporary guidelines, namely a 24-h mean systolic blood pressure ≥130 mmHg and/or a 24-h mean diastolic blood pressure ≥80 mmHg [4,5]. Participants not meeting these thresholds were classified as non-hypertensive.

### 2.5. Clinical and Laboratory Assessments

Demographic, anthropometric, and lifestyle characteristics were assessed using structured questionnaires. Height and weight were measured using a stadiometer and a calibrated digital scale, respectively, with weight recorded to the nearest 0.1 kg. During height measurement, participants stood in the Frankfurt plane. Body mass index (BMI) was calculated as weight (kg)/height (m^2^) and classified as normal weight (<25.0 kg/m^2^), overweight (25.0–29.9 kg/m^2^), or obese (≥30.0 kg/m^2^). Smoking status was categorized as current smoker or non-smoker, and regular exercise was defined as self-reported physical activity performed at least 150 min weekly during the previous 3 months. In addition to the DASH-Q, two supplementary questions were used to characterize salt-related dietary behaviors: the frequency of adding salt to food before tasting and the frequency of consuming salty snacks after 8:00 PM during the previous month. These supplementary items were used as descriptive and covariate measures reflecting discretionary salt-related behaviors only and were not intended to estimate total sodium intake or to substitute for objective sodium or potassium assessment.

Fasting venous blood samples collected at outpatient admission were analyzed for serum glucose, total cholesterol, low-density lipoprotein (LDL) cholesterol, high-density lipoprotein (HDL) cholesterol, triglycerides, urea, creatinine, uric acid, albumin, C-reactive protein (CRP), aspartate aminotransferase (AST), and alanine aminotransferase (ALT) using standard automated laboratory methods. All laboratory parameters were obtained from routine clinical biochemical analyses, and no additional tests were requested for research purposes.

### 2.6. Statistical Analysis

Continuous variables were assessed for normality using the Shapiro–Wilk test and visual inspection of histograms and Q–Q plots. Normally distributed continuous variables are presented as mean ± standard deviation (SD), and non-normally distributed variables are presented as median (interquartile range [IQR]). Categorical variables are presented as frequencies (*n*) and percentages (%). Between-group comparisons according to ABPM-defined hypertension status were performed using the independent-samples t-test or Mann–Whitney U test for continuous variables and Pearson’s chi-square test for categorical variables, as appropriate. Differences in anthropometric and biochemical parameters across DASH-Q categories were assessed using one-way analysis of variance (ANOVA) or the Kruskal–Wallis test, depending on the distribution of each variable. For variables with significant overall differences, post hoc pairwise comparisons were conducted using the Games–Howell test for normally distributed variables and Bonferroni-adjusted comparisons for non-normally distributed variables. Bivariate associations between 24-h mean SBP and DBP and dietary and biochemical parameters were evaluated using Pearson or Spearman correlation analysis, according to the distribution of each variable. Two multivariable linear regression models were constructed using the Enter method to assess the independent associations of DASH-Q total score with 24-h mean SBP and DBP, respectively. Both models were adjusted for age, sex, BMI, smoking status, regular physical activity, and frequency of adding salt before tasting. Unstandardized regression coefficients (B), standardized coefficients (β), 95% confidence intervals (CI), and variance inflation factors (VIF) were reported to assess effect size and multicollinearity, respectively. A binary logistic regression model was constructed using the Enter method to evaluate whether DASH-Q total score was independently associated with the odds of ABPM-defined hypertension, with the same set of covariates as in the linear regression models. Model fit was assessed using the omnibus chi-square test and Nagelkerke R^2^. Calibration was evaluated using the Hosmer–Lemeshow goodness-of-fit test, with *p* > 0.05 indicating adequate calibration. Classification accuracy was reported as overall percentage correctly classified, sensitivity, and specificity at a probability threshold of 0.50. In sensitivity analyses, analysis of covariance (ANCOVA) adjusted for age, sex, and BMI was used to examine whether the between-group differences in lipid profile, glycaemia, and inflammatory markers observed across DASH-Q categories persisted after covariate adjustment. The homogeneity of error variances assumption was evaluated using Levene’s test. Where this assumption was violated, results were interpreted with appropriate caution given that ANCOVA is generally robust to this violation when group sizes are not severely unequal. Statistical significance was set at a two-tailed *p* < 0.05 for all analyses. All statistical analyses were performed using IBM SPSS Statistics, version 24.0 (IBM Corporation, Armonk, NY, USA).

## 3. Results

Table 1 summarizes the baseline characteristics of the study population according to 24-h ABPM-defined hypertension status. The mean age of the participants was 55.24 ± 13.73 years, and 54.2% were female. Age, sex distribution, body mass index, BMI category, smoking status, regular exercise, and consumption of salty snacks after 8:00 PM did not differ significantly between participants with and without hypertension (all *p* > 0.05). In contrast, the frequency of adding salt before tasting food differed significantly according to hypertension status (*p* = 0.020), with a lower proportion of participants reporting that they never added salt before tasting in the hypertension group. With respect to laboratory parameters, participants with hypertension had significantly higher LDL cholesterol, triglyceride, glucose, and C-reactive protein levels, and significantly lower HDL cholesterol levels than those without hypertension (all *p* < 0.05). Urea, uric acid, creatinine, albumin, AST, ALT, and total cholesterol levels were comparable between the groups (all *p* > 0.05), although uric acid showed a borderline difference (*p* = 0.057). Consistent with the study design, both 24-h mean systolic and diastolic blood pressure values were markedly higher in the hypertension group than in the non-hypertension group (both *p* < 0.001). In addition, DASH-Q total score was significantly lower among participants with hypertension (37.57 ± 8.58 vs. 48.89 ± 7.46, *p* < 0.001). Consistently, DASH-Q category distribution differed significantly between the groups (*p* < 0.001), with low diet quality being more frequent and high diet quality being less frequent among participants with hypertension.

Table 2 presents the comparison of anthropometric and biochemical parameters across DASH-Q categories. BMI, urea, uric acid, creatinine, albumin, AST, and ALT did not differ significantly according to diet quality (all *p* > 0.05). In contrast, LDL cholesterol, HDL cholesterol, triglycerides, glucose, C-reactive protein, and total cholesterol showed significant between-group differences. Post hoc analyses showed a graded pattern for LDL cholesterol across DASH-Q categories, with the highest levels observed in the low diet quality group and the lowest levels in the high diet quality group (all pairwise comparisons significant). HDL cholesterol was significantly higher in the high diet quality group than in the low- and moderate-quality groups, whereas the difference between the low- and moderate-quality groups was not significant. Triglyceride levels also differed across all three groups, showing the highest values in the low diet quality group and the lowest values in the high diet quality group. For glucose and C-reactive protein, the high diet quality group had significantly lower values than both the low- and moderate-quality groups, while the latter two groups did not differ significantly from each other. Total cholesterol differed significantly only between the low and moderate diet quality groups. Overall, these findings indicate that poorer DASH diet quality was associated with a less favorable metabolic profile, characterized by higher LDL cholesterol, triglycerides, glucose, C-reactive protein, and total cholesterol levels, whereas higher diet quality was associated with higher HDL cholesterol levels. Both 24-h systolic and diastolic blood pressure differed significantly across DASH-Q categories (both *p* < 0.001). For both parameters, values were progressively lower across DASH-Q categories, from the low to the moderate and high diet quality groups, and all pairwise comparisons were significant in Games–Howell post hoc analyses.

In sensitivity analyses, the between-group differences in LDL cholesterol, HDL cholesterol, triglycerides, fasting glucose, and C-reactive protein across DASH-Q categories remained statistically significant after adjustment for age, sex, and BMI using ANCOVA (LDL: F[2,221] = 11.926, *p* < 0.001, partial η^2^ = 0.097; HDL: F[2,221] = 12.065, *p* < 0.001, partial η^2^ = 0.098; triglycerides: F[2,221] = 14.525, *p* < 0.001, partial η^2^ = 0.116; glucose: F[2,221] = 7.480, *p* = 0.001, partial η^2^ = 0.063; C-reactive protein: F[2,221] = 9.884, *p* < 0.001, partial η^2^ = 0.082), suggesting that the observed metabolic differences across DASH-Q categories were not attributable to between-group variation in these covariates. The homogeneity of error variances assumption was satisfied for LDL cholesterol, HDL cholesterol, and C-reactive protein (all Levene’s test *p* > 0.05). For triglycerides and glucose, this assumption was not met (Levene’s test *p* < 0.001 and *p* = 0.002, respectively), consistent with the non-normal distributions of these variables noted in the primary analyses; however, as ANCOVA is generally robust to this violation when group sizes are not severely unequal, these findings are reported with appropriate caution.

Table 3 shows the correlations of 24-h mean systolic and diastolic blood pressure with dietary and biochemical parameters. DASH-Q total score was inversely correlated with both systolic and diastolic blood pressure (both *p* < 0.001). LDL cholesterol, triglycerides, glucose, and C-reactive protein were positively correlated with both blood pressure measures, whereas HDL cholesterol was negatively correlated with both. Urea was positively correlated only with systolic blood pressure, and creatinine only with diastolic blood pressure. No significant correlations were observed for uric acid, albumin, AST, ALT, or total cholesterol.

To identify independent predictors of 24-h ambulatory blood pressure, two separate multivariable linear regression models were constructed using the Enter method, with 24-h mean SBP and DBP as the respective dependent variables (Table 4). Both models included DASH-Q total score as the primary exposure variable, along with age, sex, BMI, smoking status, regular physical activity, and frequency of adding salt before tasting as covariates. Multicollinearity was assessed using variance inflation factors (VIF); all VIF values were below 2.0, indicating no meaningful multicollinearity among the included predictors. The SBP model was statistically significant overall and explained 37.2% of the variance in 24-h mean systolic blood pressure (adjusted R^2^ = 0.352; F[7,219] = 18.543, *p* < 0.001; Table 4). Among all variables entered, DASH-Q total score was the only statistically significant independent predictor (B = −1.068, 95% CI: −1.270 to −0.866; β = −0.589; *p* < 0.001), such that each one-unit higher DASH-Q score was associated with a 1.07 mmHg lower 24-h mean SBP after adjustment for all covariates. None of the remaining covariates—including age, sex, BMI, smoking status, regular exercise, or frequency of adding salt before tasting—were independently associated with 24-h mean SBP in the adjusted model (all *p* > 0.05). Similarly, the DBP model was statistically significant overall and accounted for 24.3% of the variance in 24-h mean diastolic blood pressure (adjusted R^2^ = 0.219; F[7,219] = 10.059, *p* < 0.001). DASH-Q total score was again the sole statistically significant independent predictor (B = −0.560, 95% CI: −0.706 to −0.414; β = −0.470; *p* < 0.001), with each one-unit higher DASH-Q score being associated with a 0.56 mmHg lower 24-h mean DBP, independently of all other covariates. No other covariate reached statistical significance in the DBP model (all *p* > 0.05).

Collectively, these findings indicate that higher DASH-Q score was independently and inversely associated with both 24-h ambulatory SBP and DBP in this treatment-naive population, after adjustment for major demographic, anthropometric, and lifestyle-related confounders. The standardized coefficients (β = −0.589 for SBP and β = −0.470 for DBP) identified DASH-Q score as the strongest correlate of ambulatory blood pressure in both models, with a comparatively larger magnitude of association observed for systolic than for diastolic blood pressure (Table 4).

To examine whether DASH diet quality was independently associated with the likelihood of ABPM-defined hypertension, a binary logistic regression model was constructed using the Enter method (Table 5), with hypertension status as the dependent variable (0 = no hypertension, 1 = hypertension). The model included DASH-Q total score as the primary predictor of interest, along with age, sex, BMI, smoking status, regular physical activity, and frequency of adding salt before tasting as covariates. The overall model was statistically significant (omnibus χ^2^[7] = 93.552, *p* < 0.001), with a Nagelkerke R^2^ of 0.450 indicating a moderately strong model fit. Calibration was satisfactory, as indicated by a non-significant Hosmer–Lemeshow test (χ^2^[8] = 14.035, *p* = 0.081). The model correctly classified 79.3% of participants overall, with a sensitivity of 78.9% and a specificity of 79.6%. Among all predictors entered, DASH-Q total score was the only statistically significant independent predictor of ABPM-defined hypertension (B = −0.158; OR = 0.854, 95% CI: 0.820–0.890; *p* < 0.001; Table 5), such that each one-unit higher DASH-Q score was associated with 14.6% lower odds of ABPM-defined hypertension after adjustment for all covariates. Regular physical activity showed a borderline and counterintuitive association with hypertension (OR = 2.009, 95% CI: 1.000–4.036; *p* = 0.050), which likely reflects selection bias inherent to this clinical referral population and should be interpreted with caution. Age, sex, BMI, smoking status, and frequency of adding salt before tasting were not independently associated with hypertension status (all *p* > 0.05). Collectively, the results of the logistic regression analysis corroborate the findings of the linear regression models and confirm that higher DASH-Q score was independently and consistently associated with lower ambulatory blood pressure values and lower odds of ABPM-defined hypertension across all primary analyses.

## 4. Discussion

The present cross-sectional study demonstrated a graded inverse association between DASH diet quality, assessed by the validated DASH-Q questionnaire, and 24-h ambulatory blood pressure in treatment-naive adults referred for diagnostic ABPM. Higher DASH-Q scores were associated with lower 24-h mean systolic and diastolic blood pressure across all analytical approaches, including unadjusted group comparisons, bivariate correlation analyses, and multivariable linear regression. Furthermore, higher DASH-Q score was independently associated with lower odds of ABPM-defined hypertension in binary logistic regression, with each one-unit higher DASH-Q score corresponding to 14.6% lower odds of hypertension after adjustment for major confounders.

These findings are consistent with the established body of evidence supporting the blood pressure-lowering effects of the DASH diet. The original DASH trial demonstrated that the DASH dietary pattern lowered systolic blood pressure by approximately 5.5 mmHg and diastolic blood pressure by 3.0 mmHg compared with a typical American diet [7]. The subsequent DASH-Sodium trial further showed an additive blood pressure-lowering effect when the DASH pattern was combined with sodium restriction [8]. Multiple systematic reviews and meta-analyses have since confirmed these effects across different populations and clinical settings, with pooled reductions ranging from approximately 3 to 11 mmHg for systolic and 2 to 6 mmHg for diastolic blood pressure [9,10,11,12]. The present study extends this literature by focusing on ambulatory rather than office-based blood pressure and by employing a dedicated DASH-oriented diet quality instrument, thereby addressing two methodological gaps identified in prior observational research.

A key methodological strength of the present study is the use of 24-h ABPM for blood pressure assessment. ABPM provides a more comprehensive estimate of blood pressure burden than office measurement, minimizing white-coat effects and capturing the full circadian blood pressure profile, including nocturnal values [13,14,15]. Landmark studies have demonstrated that ambulatory blood pressure outperforms office blood pressure in predicting cardiovascular events and mortality [13,16], and current international guidelines accordingly recommend ABPM as the preferred method for confirming hypertension diagnosis and for out-of-office risk stratification [4,5,17]. While most prior studies evaluating dietary patterns and blood pressure have relied on clinic measurements, the present study provides an ABPM-based estimate of the relationship between DASH diet quality and ambulatory blood pressure burden.

Another important strength is that the study exclusively enrolled treatment-naive participants undergoing evaluation for a suspected new diagnosis of hypertension. This design avoids confounding by antihypertensive medication and allows the diet–blood pressure relationship to be examined before pharmacological modification of blood pressure. Accordingly, the present findings may be particularly relevant to the early diagnostic phase of hypertension management, before pharmacological intervention is initiated.

The standardized regression coefficients observed in the adjusted linear regression models (β = −0.589 for SBP and β = −0.470 for DBP), together with the unstandardized estimate of approximately 1.07 mmHg lower 24-h SBP per one-point higher DASH-Q score, indicate a relatively large association between DASH-Q score and ambulatory blood pressure. When extrapolated across a 10-point difference in DASH-Q score, this magnitude would exceed the average blood pressure reductions reported in several DASH intervention trials and meta-analyses [7,8,9,10,11], and therefore should be interpreted cautiously rather than as a direct causal effect. Several factors may have amplified the observed association, including residual confounding by unmeasured socioeconomic, educational, health-awareness, or broader lifestyle factors; referral bias within a clinically selected population undergoing diagnostic ABPM; reverse causality, whereby individuals concerned about elevated blood pressure may have improved their diet before evaluation; and characteristics of the DASH-Q scoring system, which may capture a broader cluster of health-oriented behaviors rather than isolated dietary exposure alone. Therefore, the observed effect sizes should be regarded as association estimates within this referral sample and not as expected blood pressure reductions achievable through DASH improvement. The pronounced gradients observed across DASH-Q categories—with 89.8% of participants in the low diet quality group meeting ABPM-defined hypertension thresholds compared with 15.2% in the high diet quality group—are consistent with a clinically meaningful diet–blood pressure relationship but also underscore the need for confirmation in prospective and interventional study designs. Nonetheless, the consistency of findings across multiple complementary analytical approaches—including group comparisons, correlation analyses, multivariable linear regression, logistic regression, and ANCOVA-based sensitivity analyses—supports the internal robustness of the observed pattern.

The logistic regression analysis adds a clinically relevant dimension to the present findings by framing the diet–blood pressure relationship in terms of a categorical clinical outcome. An adjusted odds ratio of 0.854 per one-unit DASH-Q increment corresponds to a substantial reduction in the likelihood of ABPM-defined hypertension across the observed score range. To our knowledge, few observational studies have evaluated the association between DASH diet quality and ambulatory hypertension status as a binary outcome using a validated DASH-specific instrument. This approach complements the continuous blood pressure analyses and may help contextualize the observed association during the diagnostic evaluation phase.

An additional finding of interest is the association between DASH diet quality and metabolic profile observed in the present study. Participants with higher DASH-Q scores had significantly lower LDL cholesterol, triglyceride, glucose, and C-reactive protein levels, and higher HDL cholesterol levels, with these differences persisting after adjustment for age, sex, and BMI in sensitivity analyses. These results are consistent with prior evidence demonstrating favorable effects of DASH dietary adherence on lipid profiles and markers of systemic inflammation [23], and suggest that the cardiovascular benefits of the DASH pattern may extend beyond blood pressure to encompass broader cardiometabolic risk reduction. Whether these metabolic differences mediate, moderate, or are simply co-associated with the observed blood pressure differences cannot be determined from the present cross-sectional data, and this question warrants investigation in future longitudinal research. Although BMI did not differ significantly across DASH-Q categories, waist-to-hip ratio data were unavailable; therefore, we could not determine whether unmeasured differences in central adiposity may have contributed to the observed differences in glucose, triglycerides, and C-reactive protein.

The present findings are consistent with current international guideline recommendations that emphasize the DASH dietary pattern as a first-line nonpharmacological strategy for the prevention and management of elevated blood pressure [4,5,6,24]. The 2024 ESC guidelines specifically advocate comprehensive lifestyle interventions—including dietary modification, sodium reduction, and increased potassium intake—as foundational elements of blood pressure management [5]. In this context, the present results suggest that overall DASH diet quality may be clinically relevant even during the diagnostic evaluation phase, prior to the initiation of antihypertensive therapy. At the same time, the present data should not be interpreted as evidence of causality, and the observed associations should be regarded as hypothesis-supporting rather than treatment-establishing findings.

The absence of significant between-group differences in BMI, smoking, regular exercise, and evening salty snack consumption between hypertensive and non-hypertensive participants is noteworthy and suggests that the association between DASH-Q score and ambulatory blood pressure was not simply explained by these measured lifestyle factors. However, the salt-related questions used in this study captured only self-reported discretionary salt-related behaviors and were not designed to quantify total daily sodium or potassium intake. The lack of an independent association between adding salt before tasting and ambulatory blood pressure in the adjusted models should therefore not be interpreted as evidence that sodium exposure was unrelated to blood pressure. This null finding may reflect the limited precision of a single self-reported salt-adding behavior, possible underreporting or misclassification, and the absence of objective sodium and potassium assessment, such as 24-h urinary sodium and potassium excretion. Accordingly, adjustment for adding salt before tasting should not be interpreted as adequate control for dietary sodium exposure. Future studies should incorporate objective measures of sodium and potassium exposure—such as 24-h urinary sodium and potassium excretion—alongside more comprehensive dietary assessment to better characterize the contribution of sodium and potassium to the diet–blood pressure relationship.

Several mechanisms may underlie the inverse association between DASH diet quality and blood pressure. The DASH dietary pattern is characterized by high intakes of potassium, magnesium, calcium, and dietary fiber, alongside lower saturated fat intake; when combined with sodium reduction, these features have established roles in blood pressure regulation [7,8,25,26]. Potassium promotes natriuresis and vasodilation, while magnesium and calcium contribute to vascular smooth muscle relaxation [24]. Additionally, the anti-inflammatory and antioxidant properties of the DASH pattern may improve endothelial function and attenuate arterial stiffness, further contributing to the observed blood pressure differences [26,27]. These mechanisms are biologically plausible; however, the present cross-sectional study was not designed to evaluate mechanistic pathways directly, and causal conclusions cannot be drawn from the available data.

### Limitations

This study has several limitations that should be acknowledged. First, the cross-sectional design precludes causal inference, and the possibility of reverse causation cannot be excluded. Second, the DASH-Q is a self-report instrument susceptible to recall and social desirability bias, although the validated Turkish version has demonstrated acceptable psychometric properties [19]. As with all questionnaire-obtained dietary data, DASH-Q responses may be affected by inaccurate portion or frequency reporting, selective recall, and participants’ tendency to report healthier dietary behaviors than they actually practice. In addition, the DASH-Q provides a brief estimate of DASH-oriented diet quality rather than a detailed quantitative assessment of nutrient intake, total energy intake, or actual food consumption. Therefore, misclassification of DASH adherence cannot be excluded. Third, the study did not include assessment of total energy intake, individual macronutrient composition, detailed dietary sodium intake, or 24-h urinary sodium and potassium excretion. Therefore, sodium and potassium exposure could not be adequately quantified, and adjustment for self-reported salt-adding behavior should not be interpreted as comprehensive control for sodium intake. Fourth, important potential confounders—including socioeconomic status, education level, and psychological factors—were not captured in the adjusted models. In addition, waist and hip circumference were not systematically recorded; therefore, waist-to-hip ratio could not be calculated. Because BMI does not fully capture central adiposity, the absence of waist-to-hip ratio limits our ability to determine whether the observed differences in glucose, triglycerides, and C-reactive protein were partly related to differences in abdominal fat distribution. Fifth, the single-center design and the specific characteristics of a cardiology outpatient referral population may limit the generalizability of findings to other clinical settings and population groups; the unexpected borderline association between regular exercise and hypertension observed in the logistic regression model is likely to reflect referral bias in this population and should not be generalized. Finally, the large explained variance and standardized coefficients observed in the adjusted models are notably higher than those typically reported in comparable studies and require confirmation in independent, more heterogeneous populations before broader conclusions can be drawn.

## 5. Conclusions

In conclusion, this cross-sectional study demonstrated a strong and independent inverse association between DASH diet quality and 24-h ambulatory blood pressure, as well as with the odds of ABPM-defined hypertension, in treatment-naive adults undergoing diagnostic blood pressure evaluation. Higher DASH-Q scores were consistently associated with lower ambulatory systolic and diastolic blood pressure and a more favorable metabolic profile across multiple analytical approaches. However, given the cross-sectional design, these results describe an association rather than a causal relationship, and reverse causation—for example, individuals concerned about elevated blood pressure adopting a healthier dietary pattern before evaluation—cannot be excluded. The observed associations are consistent with the broader evidence base and guideline recommendations regarding DASH-style dietary patterns in nonpharmacological blood pressure management [4,5,6,24], but should be regarded as hypothesis-generating rather than as evidence of a therapeutic effect. Prospective cohort studies and randomized controlled trials incorporating ABPM-based outcomes are needed to confirm these findings and to establish whether targeted improvement in DASH diet quality translates into clinically meaningful reductions in ambulatory blood pressure burden.

## Figures and Tables

**Table 1:** Baseline characteristics of the study population according to 24-h ABPM-defined hypertension status.

Variable	Total (*n* = 227)	No Hypertension (*n* = 113)	Hypertension (*n* = 114)	*p*-Value
Age	55.24 ± 13.73	55.19 ± 15.03	55.29 ± 12.38	0.959
Sex, *n* (%)				0.071
Female	123 (54.2)	68 (60.2)	55 (48.2)	
Male	104 (45.8)	45 (39.8)	59 (51.8)	
BMI, kg/m^2^	24.21 (23.03–26.00)	24.17 (23.04–25.78)	24.38 (23.03–26.37)	0.863
BMI category, *n* (%)				0.385
Normal weight	144 (63.4)	76 (67.3)	68 (59.6)	
Overweight	74 (32.6)	32 (28.3)	42 (36.8)	
Obese	9 (4.0)	5 (4.4)	4 (3.5)	
Smoking status, *n* (%)				0.741
No	112 (49.3)	57 (50.4)	55 (48.2)	
Yes	115 (50.7)	56 (49.6)	59 (51.8)	
Regular exercise in the last 3 months, *n* (%)				0.147
No	140 (61.7)	75 (66.4)	65 (57.0)	
Yes	87 (38.3)	38 (33.6)	49 (43.0)	
Adding salt before tasting food, *n* (%)				0.020
Never	69 (30.4)	44 (38.9)	25 (21.9)	
Sometimes	86 (37.9)	38 (33.6)	48 (42.1)	
Frequently	72 (31.7)	31 (27.4)	41 (36.0)	
Consumption of salty snacks after 8:00 PM during the last month, *n* (%)				0.351
Never	99 (43.6)	46 (40.7)	53 (46.5)	
1–2 times/month	73 (32.2)	35 (31.0)	38 (33.3)	
1–2 times/week	55 (24.2)	32 (28.3)	23 (20.2)	
LDL cholesterol, mg/dL	131.51 ± 31.51	124.00 ± 28.28	138.96 ± 33.57	<0.001
HDL cholesterol, mg/dL	46.15 ± 7.24	48.70 ± 6.81	43.62 ± 7.16	<0.001
Triglycerides, mg/dL	138.0 (108.0–222.0)	127.0 (108.0–168.0)	168.0 (108.0–264.0)	<0.001
Urea, mg/dL	29.0 (23.0–36.0)	28.0 (22.0–34.0)	29.0 (24.0–38.0)	0.162
Uric acid, mg/dL	5.23 ± 1.35	5.06 ± 1.37	5.40 ± 1.32	0.057
Glucose, mg/dL	95.0 (86.0–116.0)	91.0 (82.0–101.0)	104.5 (89.0–124.0)	<0.001
Creatinine, mg/dL	0.78 ± 0.18	0.77 ± 0.18	0.79 ± 0.18	0.312
C-reactive protein, mg/L	3.33 (1.03–4.38)	2.20 (0.92–4.12)	3.61 (1.44–4.43)	0.038
Albumin, g/L	40.24 ± 4.57	40.33 ± 4.24	40.14 ± 4.89	0.758
AST, U/L	20.0 (17.0–25.0)	20.0 (16.0–25.0)	21.0 (17.0–26.0)	0.893
ALT, U/L	19.0 (14.0–27.0)	19.0 (15.0–27.0)	18.5 (13.0–26.0)	0.380
Total cholesterol, mg/dL	217.93 ± 42.98	214.72 ± 36.98	221.11 ± 48.28	0.264
24-h SBP, mmHg	125.78 ± 17.66	109.29 ± 2.73	142.12 ± 9.25	<0.001
24-h DBP, mmHg	72.37 ± 11.51	63.21 ± 4.42	81.45 ± 9.38	<0.001
DASH-Q total score	43.20 ± 9.50	48.89 ± 7.46	37.57 ± 8.58	<0.001
DASH-Q category, *n* (%)				<0.001
Low diet quality	59 (26.0)	6 (5.3)	53 (46.5)	
Moderate diet quality	102 (44.9)	51 (45.1)	51 (44.7)	
High diet quality	66 (29.1)	56 (49.6)	10 (8.8)	

Data are presented as mean ± standard deviation for normally distributed continuous variables, median (IQR) for non-normally distributed continuous variables, and *n* (%) for categorical variables. Group comparisons were performed using the independent samples *t*-test, Mann–Whitney U test, or Pearson’s chi-square test, as appropriate. ABPM, ambulatory blood pressure monitoring; DASH-Q, Dietary Approaches to Stop Hypertension Quality questionnaire; IQR, interquartile range.

**Table 2:** Comparison of anthropometric and biochemical parameters according to DASH-Q categories.

Variable	Low Diet Quality (*n* = 59)	Moderate Diet Quality (*n* = 102)	High Diet Quality (*n* = 66)	*p*-Value
BMI, kg/m^2^	23.99 (23.03–25.60)	24.17 (23.01–25.80)	24.58 (23.55–26.70)	0.136
LDL cholesterol, mg/dL	144.90 ± 32.78 ^a^	132.48 ± 29.40 ^b^	118.05 ± 29.71 ^c^	<0.001
HDL cholesterol, mg/dL	43.36 ± 7.07 ^a^	45.73 ± 7.11 ^a^	49.30 ± 7.13 ^b^	<0.001
Triglycerides, mg/dL	203.0 (122.0–290.0) ^a^	146.0 (108.0–214.0) ^b^	116.0 (108.0–157.0) ^c^	<0.001
Urea, mg/dL	29.0 (23.0–34.0)	30.0 (24.0–40.0)	27.0 (22.0–34.0)	0.191
Uric acid, mg/dL	5.36 ± 1.28	5.35 ± 1.37	4.94 ± 1.36	0.110
Glucose, mg/dL	103.0 (87.0–124.0) ^a^	99.0 (87.0–119.0) ^a^	88.0 (80.0–97.0) ^b^	<0.001
Creatinine, mg/dL	0.77 ± 0.18	0.79 ± 0.19	0.77 ± 0.17	0.631
C-reactive protein, mg/L	3.44 (1.11–4.12) ^a^	3.68 (1.71–4.89) ^a^	1.32 (0.50–3.61) ^b^	<0.001
Albumin, g/L	40.12 ± 5.19	40.43 ± 4.59	40.04 ± 3.95	0.844
AST, U/L	21.0 (17.5–25.5)	20.0 (17.0–25.0)	21.5 (16.0–26.0)	0.691
ALT, U/L	17.0 (13.0–26.0)	19.0 (14.0–27.0)	19.5 (16.0–28.0)	0.302
Total cholesterol, mg/dL	230.64 ± 48.73 ^a^	212.77 ± 39.58 ^b^	214.53 ± 41.11 ^ab^	0.029
24-h SBP, mmHg	139.85 ± 13.61 ^a^	126.74 ± 16.53 ^b^	111.73 ± 11.45 ^c^	<0.001
24-h DBP, mmHg	79.08 ± 11.09 ^a^	73.79 ± 11.21 ^b^	64.17 ± 7.72 ^c^	<0.001

Data are presented as mean ± standard deviation for normally distributed variables and median (IQR) for non-normally distributed variables. *p* values were obtained using one-way ANOVA or the Kruskal–Wallis test, as appropriate. Different superscript letters indicate statistically significant differences between groups in post hoc pairwise comparisons; groups sharing at least one letter are not significantly different from each other. For normally distributed variables, post hoc comparisons were performed using the Games–Howell test. For non-normally distributed variables with significant overall differences, Bonferroni-adjusted pairwise comparisons were used.

**Table 3:** Correlations of 24-h mean systolic and diastolic blood pressure with dietary and biochemical parameters.

Variable	24-h Mean SBP	24-h Mean DBP
DASH-Q total score	−0.598 (<0.001)	−0.485 (<0.001)
LDL cholesterol, mg/dL	0.224 (0.001)	0.247 (<0.001)
HDL cholesterol, mg/dL	−0.335 (<0.001)	−0.172 (0.009)
Triglycerides, mg/dL	0.250 (<0.001)	0.155 (0.019)
Urea, mg/dL	0.165 (0.013)	0.090 (0.175)
Uric acid, mg/dL	0.103 (0.122)	0.103 (0.122)
Glucose, mg/dL	0.334 (<0.001)	0.218 (0.001)
Creatinine, mg/dL	0.120 (0.072)	0.133 (0.046)
C-reactive protein, mg/L	0.224 (0.001)	0.155 (0.019)
Albumin, g/L	−0.094 (0.160)	0.004 (0.952)
AST, U/L	−0.017 (0.803)	−0.014 (0.830)
ALT, U/L	−0.096 (0.150)	−0.049 (0.459)
Total cholesterol, mg/dL	−0.001 (0.992)	0.035 (0.602)

Data are presented as correlation coefficient (*p* value). Pearson correlation analysis was used for normally distributed variables, whereas Spearman’s rank correlation analysis was used for non-normally distributed variables. SBP, systolic blood pressure; DBP, diastolic blood pressure; DASH-Q, Dietary Approaches to Stop Hypertension Quality questionnaire.

**Table 4:** Multivariable linear regression analyses for 24-h ambulatory blood pressure parameters.

	24-h Mean SBP (mmHg)			24-h Mean DBP (mmHg)		
Predictor	B (95% CI)	β	*p*	B (95% CI)	β	*p*
**DASH-Q total score**	**−1.07 (−1.27;−0.87)**	**−0.589**	**<0.001**	**−0.56 (−0.71;−0.41)**	**−0.470**	**<0.001**
Sex (male vs. female)	2.49 (−1.30; 6.28)	0.070	0.197	−0.54 (−3.28; 2.20)	−0.023	0.699
Age (years)	0.05 (−0.09; 0.19)	0.039	0.474	0.00 (−0.10; 0.10)	0.000	0.996
BMI (kg/m^2^)	0.39 (−0.29; 1.08)	0.061	0.260	−0.04 (−0.53; 0.46)	−0.008	0.890
Smoking (yes vs. no)	−1.06 (−4.90; 2.79)	−0.030	0.588	−0.76 (−3.54; 2.02)	−0.032	0.590
Regular exercise (yes vs. no)	1.89 (−2.04; 5.82)	0.052	0.345	0.73 (−2.11; 3.57)	0.031	0.611
Adding salt before tasting ^a^	1.81 (−2.49; 6.11)	0.047	0.408	2.00 (−1.11; 5.10)	0.079	0.206
SBP model: R^2^ = 0.372, adjusted R^2^ = 0.352, SEE = 14.33 mmHg, F(7,219) = 18.543, *p* < 0.001 DBP model: R^2^ = 0.243, adjusted R^2^ = 0.219, SEE = 10.35 mmHg, F(7,219) = 10.059, *p* < 0.001						

B, unstandardized regression coefficient; 95% CI, 95% confidence interval; β, standardized coefficient; SBP, systolic blood pressure; DBP, diastolic blood pressure; DASH-Q, Dietary Approaches to Stop Hypertension Quality questionnaire; BMI, body mass index; SEE, standard error of the estimate. Both models were constructed using the Enter method. Multicollinearity was evaluated using variance inflation factors; all VIF values were < 2.0. DASH-Q total score is presented in bold to highlight its status as the primary exposure variable of interest. ^a^ Adding salt before tasting was dichotomized as never (0) vs. sometimes or frequently (1).

**Table 5:** Binary logistic regression analysis of independent predictors of ABPM-defined hypertension.

Predictor	B	SE	Wald χ^2^	OR (95% CI)	*p*
**DASH-Q total score**	**−0.158**	**0.021**	**55.507**	**0.854 (0.820–0.890)**	**<0.001**
Sex (male vs. female)	0.407	0.337	1.463	1.503 (0.777–2.908)	0.226
Age (years)	0.012	0.013	0.870	1.012 (0.987–1.037)	0.351
BMI (kg/m^2^)	0.069	0.063	1.191	1.071 (0.947–1.213)	0.275
Smoking (yes vs. no)	−0.376	0.348	1.165	0.686 (0.347–1.359)	0.280
Regular exercise (yes vs. no)	0.698	0.356	3.841	2.009 (1.000–4.036)	0.050
Adding salt before tasting ^a^	0.204	0.375	0.295	1.226 (0.588–2.554)	0.587
Overall model: omnibus χ^2^(7) = 93.552, *p* < 0.001|Nagelkerke R^2^ = 0.450|Hosmer–Lemeshow χ^2^(8) = 14.035, *p* = 0.081 Classification accuracy: overall 79.3%; sensitivity 78.9%; specificity 79.6%					

OR, odds ratio; CI, confidence interval; SE, standard error; DASH-Q, Dietary Approaches to Stop Hypertension Quality questionnaire; BMI, body mass index; ABPM, ambulatory blood pressure monitoring. The model was constructed using the Enter method. Model calibration was assessed using the Hosmer–Lemeshow test; a *p* value > 0.05 indicates adequate calibration. DASH-Q total score is presented in bold to denote its status as the primary predictor of interest. ^a^ Adding salt before tasting was dichotomized as never (0) vs. sometimes or frequently (1).

## Data Availability

The datasets generated and/or analyzed during the current study are not publicly available due to institutional data protection policies but are available from the corresponding author on reasonable request.

## Abbreviations

The following abbreviations are used in this manuscript:

DASH	Dietary Approaches to Stop Hypertension
ABPM	Ambulatory Blood Pressure Monitoring
DASH-Q	Dietary Approaches to Stop Hypertension Quality
BMI	Body mass index
h	Hour
TG	Triglycerides
HDL	High-density lipoprotein cholesterol
LDL	Low-density lipoprotein cholesterol
CRP	C-reactive protein
AST	Aspartate Aminotransferase
ALT	Alanine Aminotransferase
